# Diagnostic accuracy of linked color imaging and white light imaging for early gastric cancer and gastrointestinal metaplasia: a systematic review and meta-analysis

**DOI:** 10.3389/fonc.2024.1480651

**Published:** 2024-11-15

**Authors:** Hui Duan, Xinxu Zhou, Qian Li, Liu Liu, Qiong Wang, Kaiwen Wu, Lin Jiang, Xiaobin Sun

**Affiliations:** The Third People’s Hospital of Chengdu, Chengdu, Sichuan, China

**Keywords:** white light imaging (WLI), linked color imaging (LCI), early gastric cancer (EGC), gastrointestinal metaplasia (GIM), detection rate

## Abstract

**Background:**

Conventional white light imaging (WLI) frequently misses gastric cancer, resulting in a high rate of undiagnosed cases. This study compares the effectiveness of linked color imaging (LCI) and WLI in detecting early gastric cancer and gastrointestinal metaplasia, aiming to improve clinical diagnostic practices through evidence-based medical insights.

**Methods:**

The QUADAS-2 tool evaluated the quality of the studies. Additionally, methods like Split Component Synthesis (SCS) were utilized to evaluate the diagnostic performance of LCI and WLI.

**Results:**

Eleven studies involving a total of 7836 patients were included in the meta-analysis. Comparative analysis revealed that LCI demonstrated a statistically significant superiority over WLI in terms of the detection rates of EGC and GIM (detection rate of EGC: LCI vs WLI, 85% vs. 56.7%, p=0.004, OR 4.78, 95% CI 2.33-9.82, I2 = 71%; detection rate of GIM: LCI vs WLI, 88.9% vs. 40.1%, p=0.0003, OR 9.94, 95% CI 5.59-17.68, I2 = 71%). Additionally, LCI exhibited better sensitivity and specificity for the diagnosis of EGC and GIM compared to WLI. For the entire cohort, the sensitivity of LCI for EGC detection was 80% (95% CI 71%-86%) with a specificity of 82% (95% CI 63%-92%), while for GIM detection, the sensitivity was 87% (95% CI 81%-92%) with a specificity of 85% (95% CI 77%-91%).

**Conclusions:**

The detection efficiency of LCI for EGC and GIM is better than that of WLI, and LCI is recommended as the main screening method for EGC and GIM.

**Systematic review registration:**

https://www.crd.york.ac.uk/prospero/, identifier CRD42023452140.

## 1 Introduction

Globally, gastric cancer ranks fourth in mortality among all malignant tumors (1). The five-year survival rate for advanced gastric cancer is merely 15-25%, while that for Early Gastric Cancer (EGC) exceeds 90%. Therefore, early detection and treatment are pivotal for improving the survival and quality of life of gastric cancer patients (2–6). EGC and precancerous lesions typically lack obvious clinical symptoms and may manifest with atypical symptoms like loss of appetite and abdominal discomfort. By the time overt symptoms arise, the disease often progresses to advanced stages. Thus, endoscopic screening for gastric mucosa-related diseases is imperative to enhance prognosis and reduce the medical and societal burden.

The detection rate of EGC and precancerous lesions directly impacts prognosis. Emerging evidence underscores the potential of new gastroscopy techniques to enhance EGC and intestinal metaplasia detection. Endoscopists worldwide are continually exploring novel methods for early detection of gastrointestinal tract cancers and precancerous lesions, such as GIM and atrophic gastritis (7). Esophagogastroduodenoscopy remains the primary diagnostic tool for EGC and precancerous lesions of the stomach, playing a pivotal role in early detection and prevention of gastric cancer in clinical practice (8). Studies have consistently highlighted the effectiveness of gastroscopy in early detection and prevention of gastric cancer (9, 10). Early detection and timely treatment significantly mitigate the risk of gastric cancer. However, despite upper gastrointestinal endoscopy, some patients develop gastric cancer during surveillance, suggesting potential missed diagnoses (9, 11, 12). The reported rate of missed diagnosis of gastric cancer using White Light Imaging (WLI) ranges from 4.6% to 25.8% (13), underscoring the inadequacy of WLI in reducing underdiagnosis of gastric cancer and precancerous lesions.

With technological advancements, Image-Enhanced Endoscopy (IEE) techniques like Narrow-Band Imaging Endoscopy (NBI) have emerged. NBI, coupled with magnification endoscopy, enhances visualization of the mucosal glandular tube and vascular structures, thereby improving diagnostic accuracy of gastric mucosa-related diseases. However, NBI images suffer from darkness and limitations in distant observation and rapid screening. Subsequently, Linked Color Imaging (LCI) was introduced as a novel image processing technology by the LASEREO system, utilizing laser beams to simultaneously intensify and subtract colors, thereby enhancing reddish and whitish colors. LCI, an extension of BLI, combines narrowband shortwave and white light, effectively displaying gastric mucosal surface structure and blood vessels, facilitating clear lesion observation at a distance and minimizing diagnostic leakage due to poor lighting (14). EGC surrounded by intestinal metaplasia has an orange-red appearance and is surrounded by purple mucosa on LCI. In general, most early-stage gastric cancers are orange-red, orange, or orange-white on LCI. LCI has gained widespread clinical adoption, with numerous studies investigating its diagnostic efficacy for EGC and gastrointestinal metaplasia. Despite increased research on LCI’s clinical diagnosis for EGC and GIM, variations in study results persist (15–20). Therefore, this meta-analysis aims to comprehensively review the latest relevant studies, investigating LCI’s sensitivity, specificity, and diagnostic significance for EGC and GIM. This study provides robust theoretical support and evidence-based medicine for clinical diagnosis of EGC and GIM.

## 2 Methods

### 2.1 Protocol and registration

This systematic review and meta-analysis is reported in accordance with the Preferred Reporting Items for Systematic Evaluation and Meta-Analysis (PRISMA) guidelines. This systematic evaluation program was registered with the Prospective International Register of Systematic Evaluations (PROSPERO) online database (PROSPERO identifier: CRD42023452140).

### 2.2 Search strategy

We conducted a literature search for English-language articles on clinical studies published from January 1, 2018, to October 1, 2023, using Web of science, EMBASE, PubMed, and the Cochrane Library databases. Non-English articles, unpublished studies, case reports, abstracts, conference abstracts and animal studies were not included. Search terms included “early gastric cancers”, “early-stage gastric cancer”, “Early Detection of gastric cancer”, “Early Detection of gastric cancer”, “gastric carcinoma stomach cancer”, “Intestinal metaplasia”, “IEE”, “linked color imaging “, “linked color imaging endoscopy”, “Image enhanced”, “Image enhanced endoscopy”, “IEE”, “LCI”, “White light”, “White light endoscope”, “white light endomicroscopy”. A complete strategy for each database can be found in the **Supplementary Materials** (**Supplementary Document S1**).

### 2.3 Eligibility criteria

We implemented predefined broad eligibility criteria and included prospective and retrospective studies to validate the diagnostic performance of WLI and LCI for EGC and gastrointestinal metaplasia when compared with gold standard biopsy histopathology.

The included criteria are as follows: (1) all patients underwent LCI and WLI; (2) true positive (TP), false positive (FP), true negative (TN), and false negative (FN) values, or values that could be calculated based on sensitivity (Se) and specificity (Sp), were reported. (3) Detection rates were reported or could be calculated from relevant data (4) The literature stated that there was a “gold standard” (pathologic examination) for confirmation of the diagnosis. Exclusion Criteria: (1) Studies published in the form of reviews, case studies, and conference papers were excluded. (2) Literature that could not specify the number of cases and lesions. (3) Articles using the same data set were considered duplicates. (4) Literature that did not specify a “gold standard”.

### 2.4 Study selection and screening

All acquired studies were transferred to EndNote X9 where duplicates were identified and eventually eliminated. To ensure that relevant studies were not overlooked, the reference lists of all eligible articles were manually reviewed.

Two reviewers (HuiDan, XingxuZhou) screened the titles and abstracts of all articles identified in the initial literature search and then extracted data from selected articles. Disagreements were resolved by consensus. For the included articles, the full text and its associated references were reviewed in their entirety.

### 2.5 Data extraction

We extracted data from each eligible study, including first author, year of publication, country, included studies, number of lesions, sensitivity, specificity, PPV, NPV, and detection rate. This was done by two independent reviewers, with third-party input if required. All data were summarized and compiled into a table accessible to all authors.

### 2.6 Quality of studies

The methodological quality of the selected diagnostic accuracy studies was assessed using the Quality Assessment Tool for Diagnostic Accuracy Studies (QUADAS-2) (Whiting et al., 2011), which was completed independently by two reviewers, with group discussion as necessary. The QUADAS-2 Quality Assessment Scale consists of four sections: (1) case selection, (2) tests to be evaluated, (3) gold standard, (4) case flow and progress. The QUADAS-2 quality assessment scale consists of: (1) selection of cases, (2) trials to be evaluated, (3) gold standard, (4) case flow and progression, and these four sections contain 10 items. The first 3 sections are evaluated in terms of clinical applicability, and the 10 items are evaluated in terms of “yes”, “no”, “no”, “no”, “no” and “no”. All 10 items were judged according to the criteria of “yes”, “no”, and “unclear”, and the corresponding risk of bias was “low”, “no”, “unclear”, and “low”. The corresponding risk of bias was “low”, “high”, “no”, and “unclear”, “high” and “unclear”, respectively. Among them, “unclear” was mainly used when the included literature did not provide detailed content and complete data, and it was difficult for the researchers to make a judgment. The risk of bias was considered low only if all items in a section were “yes”. If one item was “no”, the section might be at risk of bias; the quality evaluation chart, risk of bias and applicability evaluation summary chart were drawn by Revman 5.4 software.

### 2.7 Data analysis

The extracted data were analyzed using Review Manager 5.4 software and Stata 15 software.

For the detection rate, data were analyzed using Review Manager 5.4 software. Count data were expressed as 95% confidence interval (95% CI), and Odds risk (OR) was used to test the count data, comparing the detection rate of EGC and the detection rate of gastrointestinal metaplasia between LCI and WLI. Heterogeneity between included studies was analyzed by χ^2^ test and described by I^2^ value. Low heterogeneity between studies was suggested when I^2^ >25%, moderate heterogeneity when 50%<I^2^<75%, and high heterogeneity when I^2^ >75%, and substantial heterogeneity was generally considered to exist for I^2^ >50%. A random-effects model was used when I^2^ >50% and a fixed-effects model when I^2^ <50% (21). P < 0.05 was considered statistically significant. The symmetry of the funnel plot was used to assess publication bias, and a forest plot was used to present the results of data analysis for each group. Sensitivity analysis was then performed using Stata15 software to determine the effect of individual studies on the overall statistical difference, while the merging of I^2^ values of the remaining literature was performed after excluding the included literature one by one. If the total I^2^ value of the remaining studies was significantly lower after the exclusion of a single study compared with that before the exclusion, it means that this literature may be the source of heterogeneity of the articles, and the reasons for the heterogeneity need to be further discussed.

For specificity and sensitivity of LCI and WLI, datas were analyzed using Stata 15 software. The Spearman correlation coefficient was used to assess the threshold effect (a strong positive correlation of the Spearman coefficient suggests the possibility of a threshold effect) (22). The degree of inter-study heterogeneity was examined using the Higgins Inconsistency Index (I^2^), with a value of the I^2^ of less than 25% being regarded as low, a value of the I^2^ of 25% to 50% being regarded as moderate, and a value of the I^2^ of 50% to 75% being regarded as high. Heterogeneity between studies was considered high when the I^2^ value was between 50% and 75%, and very high when the I^2^ value was above 75%^25^. A binary generalized mixed bivariate model was used to estimate the magnitude of the combined effects, including: Summarized Sensitivity (SSEN), Specificity (SSPE), and Specificity (SSPE). Specificity (SSPE), Summarized Positive Likelihood Ratio (SPLR), Summarized Negative Likelihood Ratio (SNLR), and Diagnostic Odd Ratio (SNLR) and Diagnostic Odd Ratio (DOR). Mori diagrams and Summary Receiver Operating Characteristics (SROC) curves were plotted and Area Under Summary Receiver Operating Characteristics (AUSROC) was calculated. Publication bias was explored by Deeks’ funnel plot, and we plotted Deek’s funnel plot asymmetry test for LCI and WLI respectively. If the funnel plot showed a result of *P*≥0.05, it indicated that there was no publication bias; if the funnel plot showed a result of *P*<0.05, it indicated that there was publication bias. Fagan plots were then plotted to reflect the diagnostic value of the diagnostic methods, and the posterior probability was calculated by setting the *a priori* probability.

## 3 Results

### 3.1 Study selection

**Figure 1:**
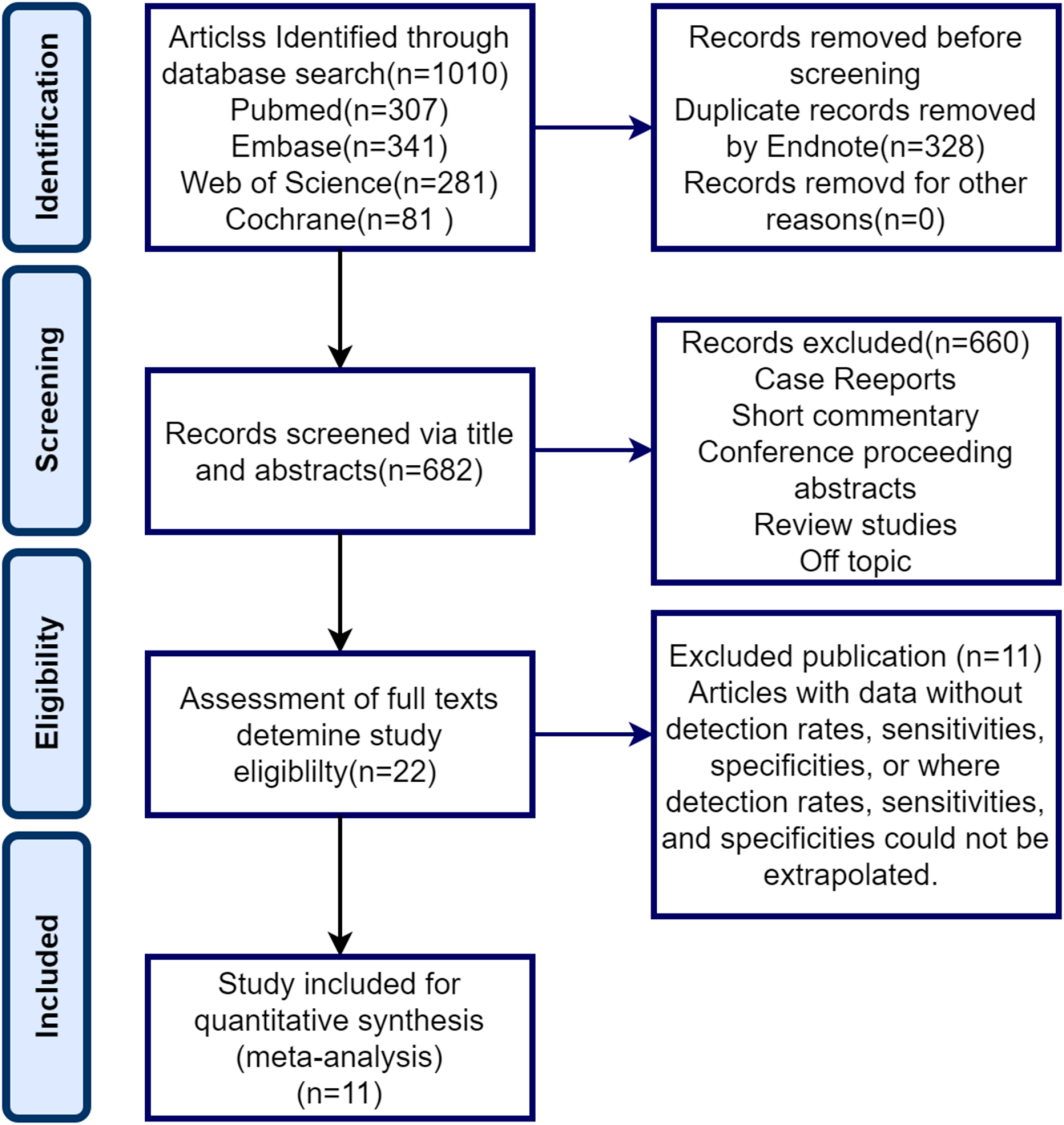
PRISMA flowchart for systematic evaluation and meta-analysis.

**Figure 1** illustrates the PRISMA flowchart, which demonstrates the process of study selection. Our exhaustive database search initially yielded 1010 articles. Of these, 328 were eliminated through EndNote and 682 were screened for titles and abstracts. Following title and abstract screening, 660 publications were excluded, leaving only 22 full-text screens. The full text of these 22 records was retrieved and reviewed for eligibility. Eleven articles were excluded for various reasons summarized in **Figure 1**, resulting in a total of 11 documents included in the analysis.

### 3.2 Study and index test characteristics

**Tables 1**, **2** show the extracted datasets of the included articles. Briefly, they were published between 2019 and 2023, all in Asian countries, with the majority from Japan (n=7), China (n=3), and Singapore (n=1).11 studies (23–33), with 3 articles retrospectively included Patients were patients with pre-existing gastrointestinal tumors and were evaluated by a specialists to evaluate past samples/images (Toshihisa 2019; Masayuki 2023; Tsevelnorov 2022);. The remaining 8 articles were prospective studies (Ken Haruma 2022; MinMin 2022; Minoru Yamaoka 2019; Shoko Ono 2021; Honglei Cheng 2019; Jiang Zhang Xiu 2021; Shoko Ono 2018; Clement 20) All articles Confirmation of the diagnosis was based on pathological findings.11 articles focused on comparing the difference between LCI and WLI in terms of detection rate, specificity, and sensitivity for early-stage cancer and gastrointestinal chemosis.

**Table 1:** Basic characteristics of the included literature and data extracted from the included studies (EGA Early gastric cancer).

Authors	Study period	Country	Inclusion of studies	Patients (n)	Indicators of outcome	
				LCI/WLI	LCI	WLI
Haruma et al. (23)	2022	Japan	Diagnostic ability of linked color imaging in ultraslim endoscopy to identify neoplastic lesions in the upper gastrointestinal tract	751/753	Detection rate89.32%(92/103)	Detection rate40.78%(42/103)
Min et al. (24)	2022	China	Diagnostic accuracy of linked color imaging versus white light imaging for early gastric cancers: a prospective, multicenter, randomized controlled trial study	914/914	1.Sensitivity 89.47% 2.Specificity78.28% 3.PPV 16.61% 4.NPV 99.35% 5.Detection rate 89.47%(51/57)	1.Sensitivity 59.65% 2.Specificity 68.95% 3.PPV 100% 4.NPV 96.71% 5.Detection rate 59.64%(34/57)
Yamaoka, et al. (25)	2019	Japan	Detection of early stage gastric cancers in screening laser endoscopy using linked color imaging for patients with atrophic gastritis	500/500	Detection rate 100%(13/13)	Detection rate 76.92%(10/13)
Ono et al. (26)	2021	Japan	Linked Color Imaging Focused on Neoplasm Detection in the Upper Gastrointestinal Tract	751/751	Detection rate 92. 31%(60/65)	Detection rate 60%(36/60 )
Khurelbaatr et al. (27)	2022	Japan	Improved detection of early gastric cancer with linked color imaging using an ultrathin endoscop e: a video-based analysis	166/166	1.Sensitivity84.00% 2.Specificity 50.60% 3.PPV 69.47% 4.NPV 70.27% 5.Detection rate 84.21%(80/95)	1.Sensitivity 69.90% 2.Specificity59.8% 3.PPV 69.94% 4.NPV 50.33% 5.Detection rate %(66/95 )
Higashino et al. (28)	2023	Japan	Improvement of detection sensitivity of upper gastrointestinal epithelial neoplasia in linked color imaging based on data of eye tracking	120/120	1.Sensitivity68.1% 2.Specificity90% 3.PPV 87.2% 4.NPV 73.9%	1.Sensitivity53.7% 2.Specificity86.7% 3.PPV 80.1% 4.NPV65.2%
Fujiyoshi et al. (29)	2019	Japan	Utility of linked color imaging for endoscopic diagnosis of early gastric cancer	43/43	1.Sensitivity 76.7% 2.Specificity93.0% 3.PPV 91.7% 4.NPV 80% 5.Detection rate 76.7%(33/43)	

**Table 2:** Basic characteristics of the included literature and data extracted from the included studies (GIM, Gastric Intestinal Metaplasia).

Authors	Study period	Country	Inclusion of studies	Patients (n)	Indicators of outcome	
				LCI/WLI	LCI	WLI
Clement (30)	2021	Singapore	A prospective randomized tandem gastroscopy pilot study of linked color imaging versus white light imaging for detection of upper gastrointestinal lesions	45/45	1.Sensitivity84.7% 2.Specificity92.2% 3.PPV76.6% 4.NPV95.2% 5.Detection rate 100%(17/17)	1.Sensitivity 32.9% 2.Specificity 97.2% 3.PPV 77.8% 4.NPV 82.7% 5.Detection rate 41.17%(7/17)
Chen et al. (31)	2019	China	Predictability of gastric intestinal metaplasia by patchy lavender color seen on linked color imaging endoscopy	107/107	1.Sensitivity90.24% 2.Specificity72.72% 3.PPV67.27% 4.NPV92.31% 5.Detection rate 36.59%(33/37)	1.Sensitivity36.59% 2.Specificity42.42% 3.PPV28.3% 4.NPV51.85% 5.Detection rate 89.18%(15/41)
Ono et al. (32)	2018	Japan	Lavender Color in Linked Color Imaging Enables Noninvasive Detection of Gastric Intestinal Metaplasia	128/128	1.Sensitivity91.40% 2.Specificity 87.10% 3.PPV85.45% 4.NPV 92.44% 5.Detection rate91.38%(53/58)	1.Sensitivity 21.20% 2.Specificity 99.20% 3.PPV95.64% 4.NPV60.31% 5.Detection rate20.69%(12/58)
Xiu et al. (33)	2021	China	Comparison between the Linked Color and White Light Imaging Combined Score in the Evaluation of High-Risk Population of Gastric Cancer	392/392	1.Sensitivity 80.7% 2.Specificity 85.3% 3.PPV 79.3% 4.NPV 86.4%	1.Sensitivity 69.6% 2.Specificity 74.9% 3.PPV 61.5% 4.NPV 82.4%

Seven articles evaluated the detection rates of Linked Color Imaging (LCI) and White Light Imaging (WLI) in early-stage cancer, while four articles assessed the detection rates of LCI and WLI in gastrointestinal metaplasia. Additionally, four articles examined the specificity and sensitivity of LCI in detecting early cancers, while another four articles assessed the specificity and sensitivity of both LCI and WLI in detecting gastrointestinal metaplasia. Ultimately, seven articles (n=7) were included in the analysis of detection rates for early cancers, and four articles (n=4) were included in the analysis of specificity and sensitivity. Furthermore, gastrointestinal metaplasia was analyzed in four articles pertaining to detection rates, specificity, and sensitivity.

### 3.3 Quality assessment

**Figure 2:**
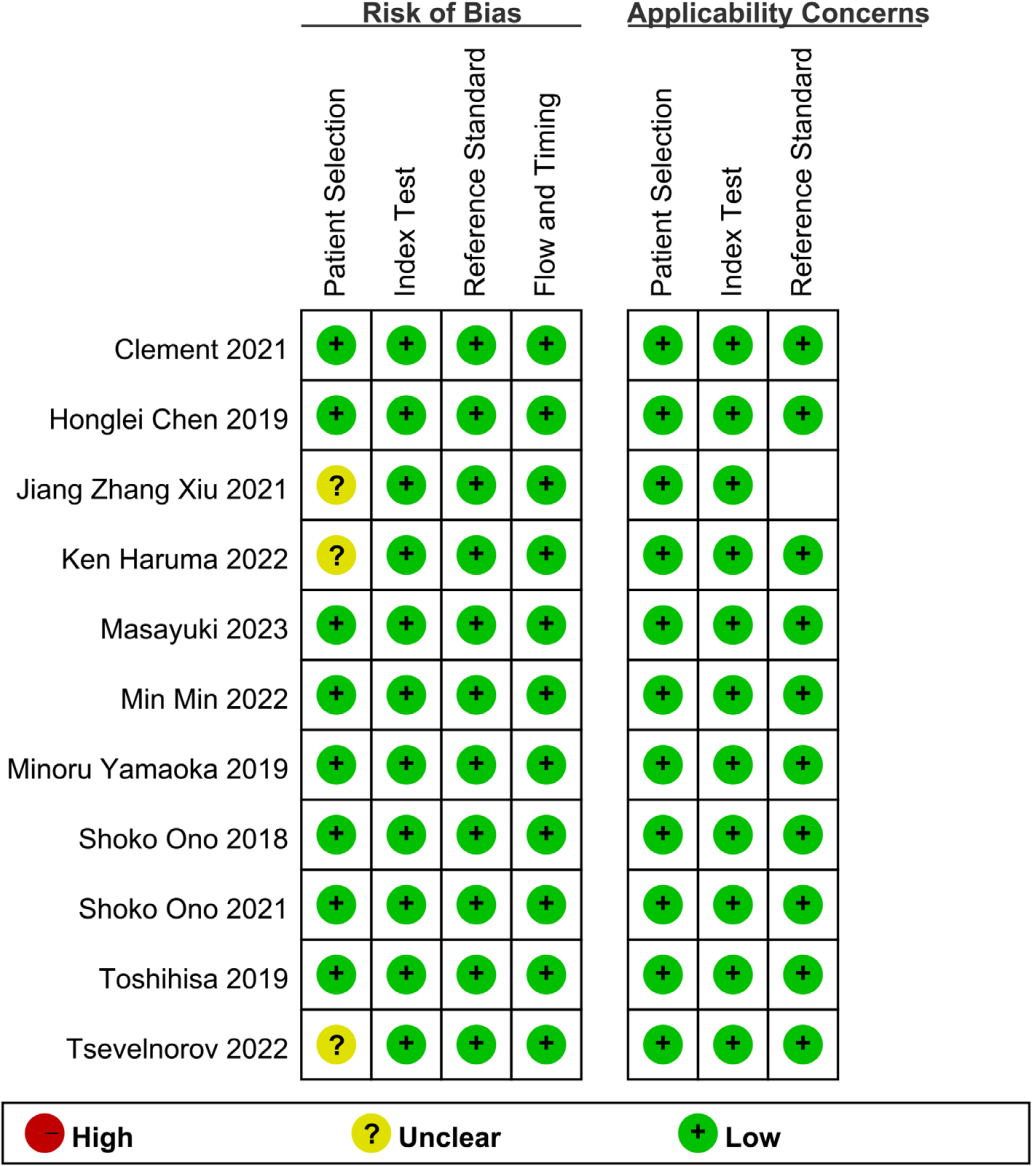
Risk of bias and applicability concerns summary.

The assessment indicates that most of the included studies did not pre-specify a threshold, leading to inconclusive answers regarding this aspect. Additionally, while the risk of bias and clinical applicability were evaluated, the overall assessment did not suggest a high-risk evaluation (**Figure 2**).

### 3.4 LCI and WLI on EGC

#### 3.4.1 Diagnostic value

**Figure 3:**
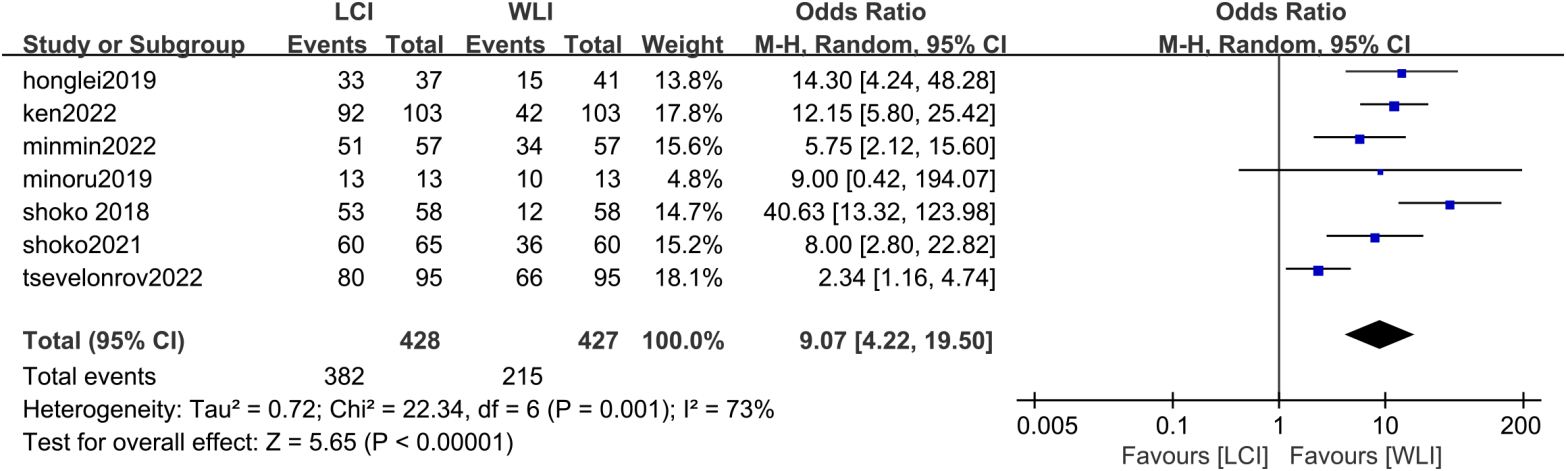
Forest plot of total GEC detection rate comparing LCI and WLI.

A total of seven studies, such as Ken Haruma 2022, contributed to the derivation of the EGC detection rate. Comparison between LCI and WLI revealed a significant improvement in the detection rate of EGC with statistical difference (OR 4.7, P < 0.0001, 95% CI 2.33-9.82, I2 = 71%) (**Figure 3**). The funnel plot displayed good symmetry and low publication bias (**Supplementary Figure S1**). Moderate heterogeneity (I^2^ > 50%) was observed in the included literature. However, sensitivity analysis demonstrated consistent results before and after exclusion, enhancing the reliability of the analysis (**Supplementary Figure S2**). Overall, LCI significantly improved the detection rate of EGC compared to WLI.

#### 3.4.2 Diagnostic efficacy

##### 3.4.2.1 Specificity and sensitivity

**Figure 4:**
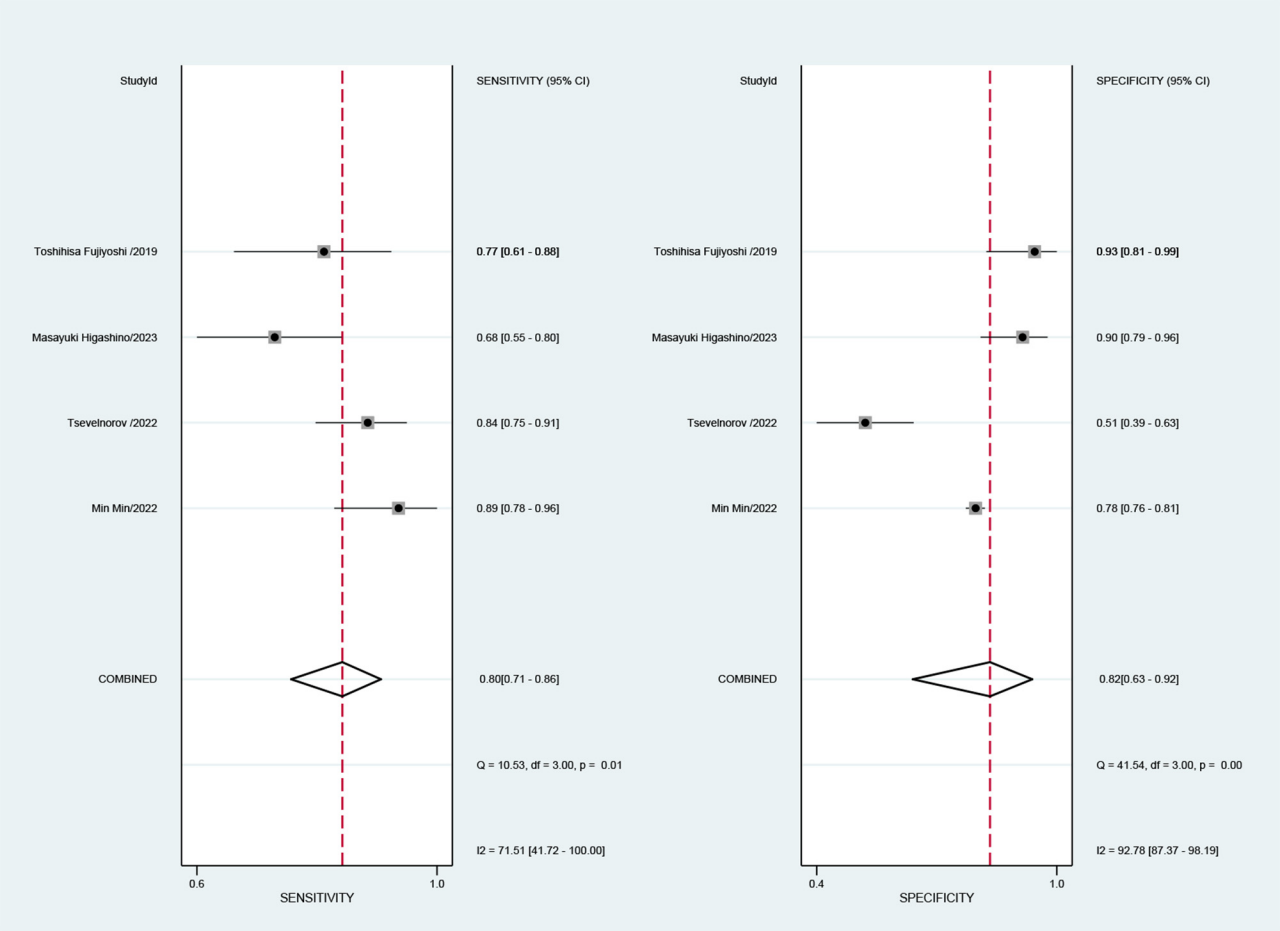
Forest plot of sensitivity and specificity of LCI for diagnosing early cancer.

**Figure 5:**
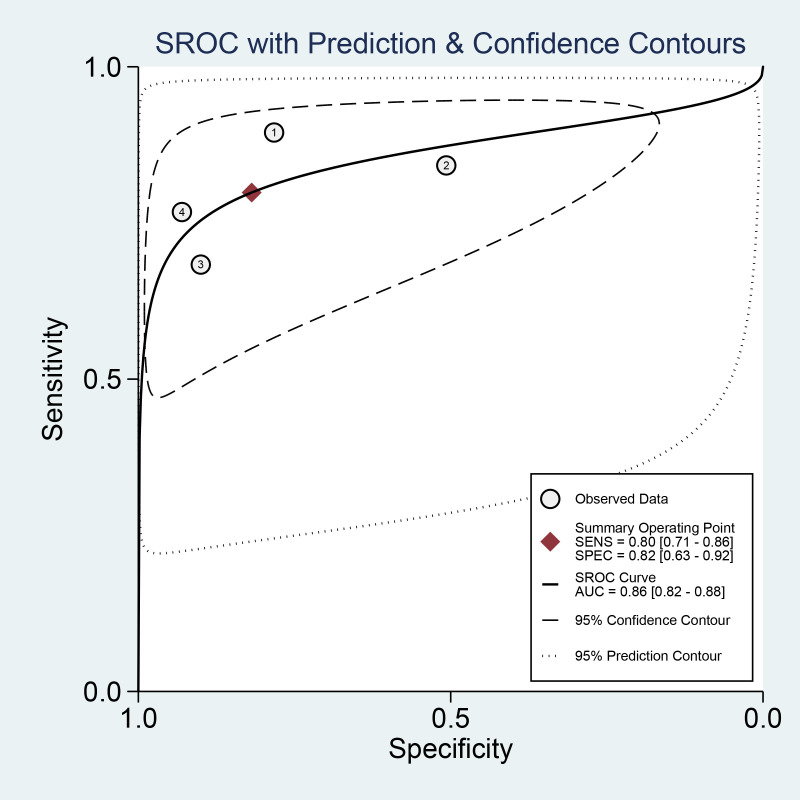
SROC curve of LCI for GEC diagnosis.

Combined results of effect sizes for LCI in diagnosing EGC across studies were as follows: sensitivity (SEN) = 0.80 (0.71-0.86), specificity (SPE) = 0.82 (0.63-0.92), positive likelihood ratio (PLR) = 4.4 (2.1-4.9), negative likelihood ratio (NLR) = 0.25 (0.18-0.33), area under the curve (AUC) = 0.86, and diagnostic odds ratio (DOR) = 18 (8-40) (**Figure 4**). Notably, no threshold effect was observed in the study, indicating homogeneity. Further, the absence of a “shoulder-wall” point distribution in the symmetrical SROD curve supported the absence of heterogeneity due to threshold effect (**Figure 5**).

##### 3.4.2.2 Publication bias

Publication bias test conducted on four papers, followed by Deek funnel plot analysis, revealed symmetric distribution around the regression line (P=0.82>0.05), indicating no statistically significant publication bias across these studies (**Supplementary Figure S3**).

##### 3.4.2.3 Clinical utility evaluation

Clinical utility evaluation using Fagan charts suggested that the application of LCI for diagnosing EGC exhibited considerable sensitivity, guiding clinical diagnosis effectively. Setting the pre-test probability at 20%, a positive LCI test resulted in a post-test probability of 52%, indicating a 52% probability of the tested person having EGC. Conversely, a negative LCI test yielded a post-test probability of 6%, signifying a 6% probability of the tested person having EGC (**Supplementary Figure S4**).

### 3.5 LCI and WLI for GIM

#### 3.5.1 Diagnostic value

**Figure 6:**
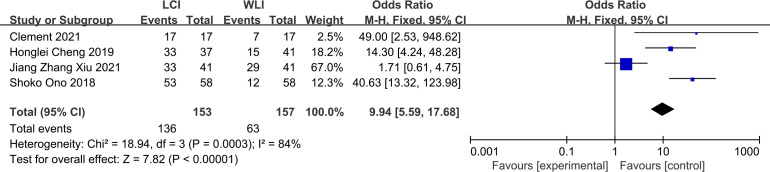
Forest plot of total GIM detection rate comparing LCI and WLI.

The total GIM detection rate was derived from four studies, such as Clement 2021, revealing a significant difference in GIM detection rate between LCI and WLI (88.8% vs. 40.1%, OR 9.94, P=0.0003, 95% CI 5.59-17.68, I2 = 84%) (**Figure 6**). The funnel plot displayed good symmetry and low publication bias (**Supplementary Figure S5**). Moderate heterogeneity (I^2^ > 50%) was observed, although sensitivity analysis reinforced the reliability of the results (**Supplementary Figure S6**). LCI notably improved the GIM detection rate compared to WLI.

#### 3.5.2 Specificity and sensitivity

##### 3.5.2.1 Combined effect sizes and heterogeneity test

**Figure 7:**
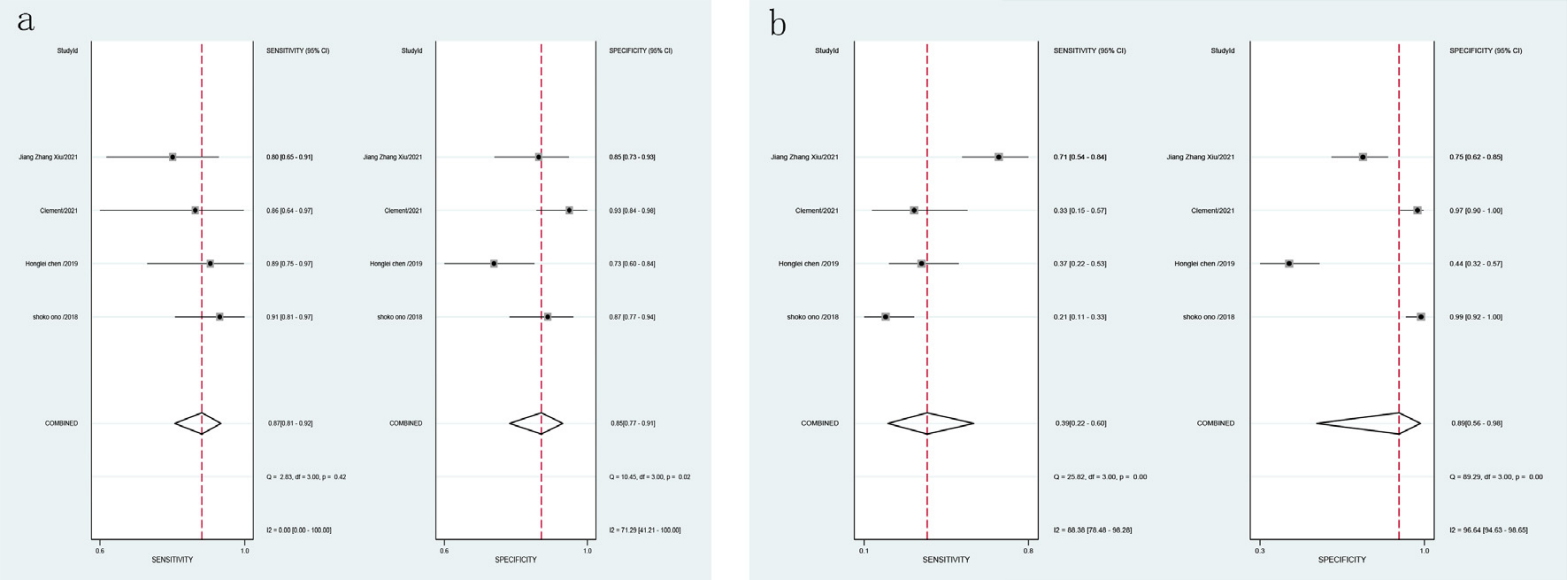
Forest plot of LCI and WLI diagnostic GIM specific sensitivity [**(A)**. Forest plot of LCI diagnostic GIM specific sensitivity; **(B)** Forest plot of WLI diagnostic specific sensitivity GIM].

**Figure 8:**
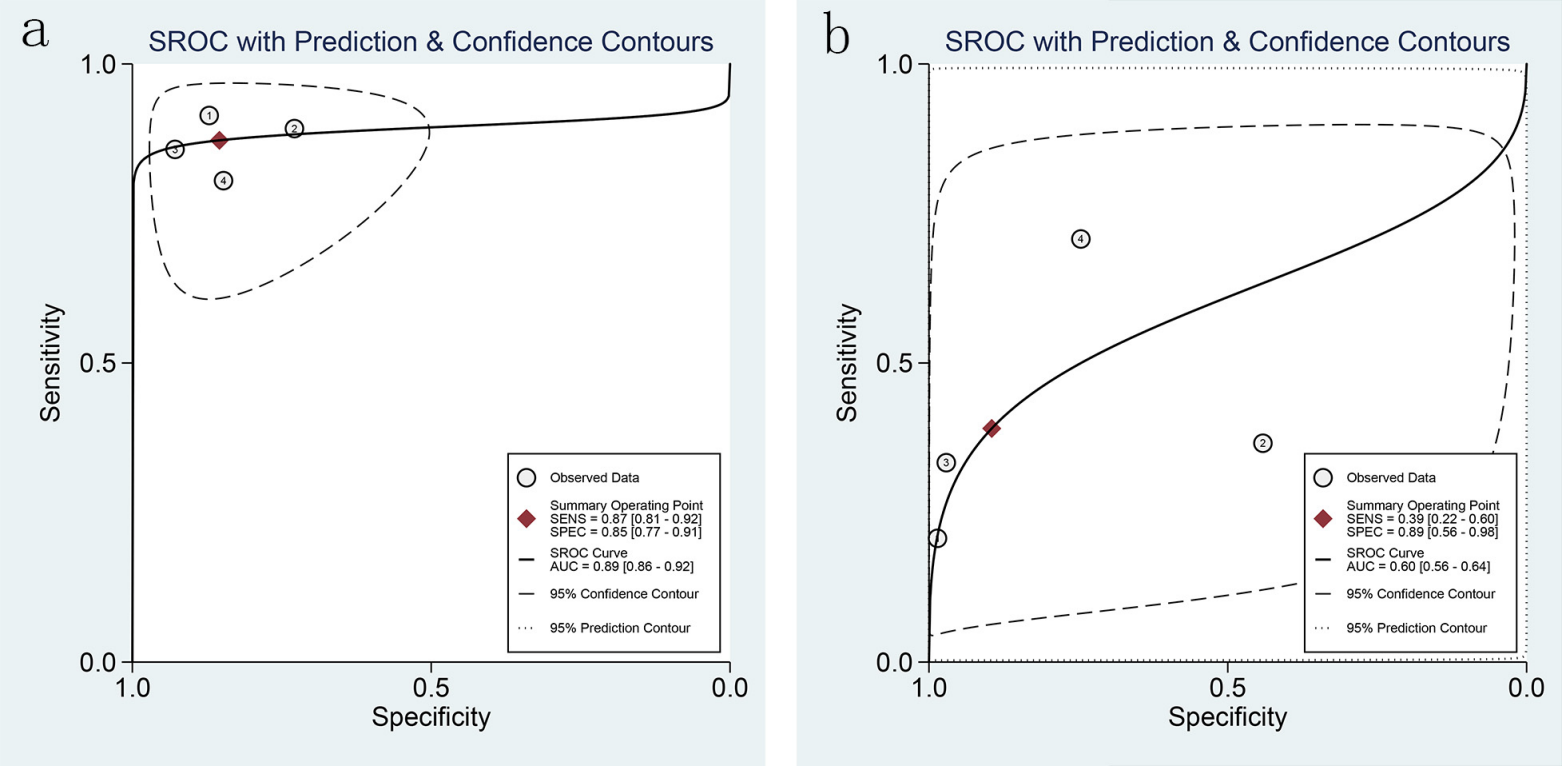
SROC curves of LCI and WLI for GIM diagnosis [**(A)**. SROC curve of LCI for GIM diagnosis; **(B)** SROC curve of WLI for GIM diagnosis].

Combined effect sizes for LCI in diagnosing GIM across studies were as follows: SEN = 0.87 (0.81-0.92), SPE = 0.85 (0.77-0.91), PLR = 6.0 (3.7-9.6), NLR = 0.15 (0.10-0.23), AUC = 0.89, and DOR = 40 (20-81). Conversely, for WLI, the effect sizes were: SEN = 0.39 (0.22-0.60), SPE = 0.89 (0.56-0.98), PLR = 3.7 (0.8-17.0), NLR = 0.68 (0.51-0.90), AUC = 0.60, and DOR = 5 (1-29) (**Figure 7**). Spearman’s correlation coefficient indicated no threshold effect in both studies, supported by symmetrical SROD curves (**Figure 8**).

##### 3.5.2.2 Publication bias

Publication bias test revealed no significant bias across the four studies (P=0.94>0.05), as indicated by the symmetric distribution in the Deek funnel plot (**Supplementary Figures S7**, **S8**).

##### 3.5.2.3 Clinical utility evaluation

Clinical utility evaluation using Fagan charts demonstrated that LCI had superior clinical applicability compared to WLI. When setting a pre-test probability of 20%, a positive LCI test resulted in a post-test probability of 60% for GIM, while a negative LCI test yielded a post-test probability of 4%. In comparison, WLI showed an average sensitivity for GIM diagnosis, resulting in a 48% probability of GIM when tested positive and a 15% probability when tested negative (**Supplementary Figures S9**, **S10**).

##### 3.5.2.4 Clinical utility evaluation

Clinical utility evaluation was conducted using Fagan charts to assess the practical application of LCI and WLI in diagnosing Gastrointestinal Metaplasia (GIM). The results suggest that LCI possesses a certain degree of sensitivity and can effectively guide clinical diagnosis of GIM.

When the pre-test probability is set at 20%, a positive LCI test yields an upper slash Positive Likelihood Ratio (PLR) of 6, resulting in a post-test probability of 60%. This indicates that there is a 60% probability of the subject having GIM when the LCI test is clinically positive for GIM. Conversely, when the pre-test probability is set at 20%, a negative LCI test results in a lower slash Negative Likelihood Ratio (NLR) of 0.15, leading to a post-test probability of 4%. This signifies a 4% probability of the subject having GIM when the LCI test is clinically negative for GIM (refer to **Supplementary Figure S9**).

In contrast, the sensitivity of applying White Light Imaging (WLI) for diagnosing gastrointestinal metaplasia is average. When setting a pre-test probability of 20%, a positive WLI test yields an upper slash PLR of 4, resulting in a 48% probability of the subject having GIM when clinically tested positive for GIM. Conversely, when setting a pre-test probability of 20% and a lower slash NLR of 0.68, the resulting post-test probability is 15%, indicating a 15% probability of the subject having GIM when clinically tested negative for GIM (refer to **Supplementary Figure S10**).

In summary, LCI demonstrates superior clinical applicability compared to WLI in diagnosing GIM, as evidenced by its higher sensitivity and greater impact on post-test probabilities.

## 4 Discussion

### 4.1 Background

White Light Imaging (WLI) technology has been instrumental in detecting gastric cancer and its precancerous lesions, significantly contributing to the diagnosis and treatment of gastric diseases since its inception. However, due to the subtle morphological manifestations and color similarities between early-stage gastric cancer (EGC) and its precancerous lesions, there is a risk of missed diagnosis, even for endoscopists who are well-versed in endoscopic practices. This potential oversight can lead to delayed diagnosis, emphasizing the need to enhance detection rates.

Historically, pigment endoscopy was employed to increase detection rates, offering superior contrast and sensitivity compared to white light imaging. However, this method presented challenges such as complex stain preparation, mucosal irritation, allergic reactions in some patients, time consumption, uneven staining, and difficulty in determining tumor depth, rendering it unsuitable for routine screening (34).

The advent of Linked Color Imaging (LCI) endoscopy has spurred continued exploration by endoscopists worldwide. Studies, including a multicenter large-sample study by Minmin et al. (24) have indicated that LCI can effectively identify EGCs during routine endoscopy by enhancing color differentiation of gastric mucosa, thereby improving detection rates. Additionally, research by Jiangzhang Xiu et al (33). suggests that LCI demonstrates higher diagnostic accuracy for intestinal metaplasia compared to WLI.

To further elucidate the potential benefits of LCI in enhancing detection rates of both EGC and GIM, we conducted a systematic meta-analysis encompassing 11 high-quality randomized controlled trials (RCTs). The analysis included a total of 6,492 patients in the EGC group and 1,344 cases in the GIM group, providing a comprehensive evaluation of whether LCI can improve detection rates for both conditions.

### 4.2 Principal findings

To the best of our knowledge, this meta-analysis represents the first comprehensive assessment of the detection rate of Linked Color Imaging (LCI) for Early Gastric Cancer (EGC) and gastrointestinal metaplasia (GIM). In this systematic review and meta-analysis, we scrutinized the accuracy of LCI and White Light Imaging (WLI) in diagnosing EGC and GIM, revealing that LCI outperformed WLI in both conditions.

In the diagnosis of EGC, LCI exhibited a detection rate of 85.75%, whereas WLI showed a detection rate of 56.70%. Similarly, in diagnosing gastrointestinal metaplasia, LCI demonstrated a detection rate of 88.89%, whereas WLI yielded a detection rate of 40.12%. Moreover, LCI displayed higher specificity and sensitivity in diagnosing both EGC and gastrointestinal metaplasia. The diagnostic efficacy of LCI for EGC was characterized by a sensitivity (SEN) of 0.80 (95% CI 0.71-0.86) and specificity (SPE) of 0.82 (95% CI 0.63-0.92), with a Positive Likelihood Ratio (PLR) of 4.4 (95% CI 2.1-4.9) and a Negative Likelihood Ratio (NLR) of 0.25 (95% CI 0.18-0.33). Similarly, for the diagnosis of gastrointestinal metaplasia, LCI demonstrated a sensitivity (SEN) of 0.87 (95% CI 0.81-0.92) and a specificity (SPE) of 0.85 (95% CI 0.77-0.91), with a PLR of 6.0 (95% CI 3.7-9.6) and an NLR of 0.15 (95% CI 0.10-0.23).In contrast, the sensitivity and specificity of WLI for diagnosing gastrointestinal metaplasia were notably lower, with a SEN of 0.39 (95% CI 0.22-0.60) and a SPE of 0.89 (95% CI 0.56-0.98), alongside a PLR of 3.7 (95% CI 0.8-17.0) and an NLR of 0.68 (95% CI 0.51-0.90).

These findings underscore the superior sensitivity and specificity of Linked Color Imaging (LCI) in diagnosing both Early Gastric Cancer (EGC) and gastrointestinal metaplasia (GIM), highlighting its enhanced diagnostic value compared to White Light Imaging (WLI). Notably, the results of this study are consistent with numerous previously published clinical studies. For instance, two large-scale, multicenter, randomized controlled trials conducted in Japan demonstrated the utility of LCI in accurately identifying gastric tumor lesions (23, 24). Additionally, studies by Haruma K (23) and Khurelbaatar T et al (27). revealed that both ultra-thin and standard LCI exhibited superior diagnostic efficacy over WLI for diagnosing EGC. Further corroborating these findings, Higashino M et al (28). illustrated that LCI surpassed WLI in sensitivity for EGC screening, particularly among novice and trainee endoscopists. Moreover, Ono S et al (26). found LCI to outperform WLI in identifying early gastric cancers in patients with atrophic gastritis. Importantly, LCI also demonstrated commendable efficacy compared to WLI in detecting GIM across several studies. Collectively, these findings suggest that LCI holds a significant advantage over WLI in the detection of both EGC and GIM, highlighting its potential as a superior diagnostic tool in clinical practice.

### 4.3 Advantages of LCI for visualization of gastric mucosal lesions

LCI, developed by Fuji in Japan in 2012, is a blue laser endoscope known as LASEREO. It emits four types of light in the pre-processing module: amber, green, white (450nm), and violet (410nm). The violet light, with its narrow wavelength, can penetrate only the surface of the mucosa. When absorbed by the surface hemoglobin, it is not reflected, but in deeper blood vessels, it is reflected. This unique property enables the differentiation of various lesions based on differences in the depth of blood vessels. By magnifying the color difference, LCI enhances the visual contrast, making whites appear whiter and reds appear redder (35). Liu Yan et al. established a color-microstructure-vessel (CMV) model based on the microscopic features of color, microstructure, and vascularity of the gastrointestinal (GI) mucosa. This model delineates three color categories: typical red (type 1), purplish-red (type 2), and purplish-ringed-yellow-red (type 3). In LCI, intestinal metaplasia appears purple, inflammation appears red, and normal mucosa or tumors appear yellow. Chronic non-atrophic gastritis, characterized by non-specific inflammation of the distal stomach, typically appears red on LCI. Gastric cancer, with its complex pathological changes, exhibits a varied color spectrum, typically showing purple ringed yellow-red hues (36).

LCI has been demonstrated to be a superior Image-Enhanced Endoscopy (IEE) technique compared to WLI because it offers better visualization of red and purple changes. This enhanced visibility makes the identification and detection of EGC or GIM easier and more accurate (30, 37–39).

### 4.4 Advantages of this study

The robustness of our findings is supported by several factors. Firstly, the substantial number of cases included in this study, totaling 7,836, ensures the reliability and representativeness of the results. Secondly, the study populations were exclusively composed of Asian individuals, minimizing potential biases related to racial classification. Additionally, all studies utilized pathological findings as the definitive criterion for judgment, ensuring consistency and accuracy in the evaluation process. Furthermore, eight of the included studies were prospective, enhancing the standardization of Randomized Controlled Trial (RCT) protocols and providing a more realistic reflection of clinical gastroenteroscopy practices. This ensures that our findings are applicable not only at a theoretical level but also in real-world clinical settings.

### 4.5 Limitation

Several limitations should be acknowledged in our study. Firstly, publication bias may have influenced the results, as studies reporting positive findings regarding the efficacy of LCI in detecting EGC and precancerous lesions might be more likely to be published. Conversely, studies with negative results may be underreported, potentially biasing the overall findings. Secondly, inherent heterogeneity among the included studies could have impacted the outcomes. Factors such as variations in bowel preparation, timing of gastroscopy, differences in endoscopists’ experience levels, and the inclusion criteria for patients (including both general patients and those at high risk of gastric cancer) may have introduced inconsistencies and affected the accuracy of our meta-analysis. Moreover, due to data limitations, we were unable to conduct subgroup analyses to assess diagnostic tests specifically for EGC and gastrointestinal metaplasia separately. Early gastric cancer may depend on the use of magnifying endoscopy and thin endoscopy, but due to the limited data in this paper we were unable to perform subgroup analyses to clarify its impact. Lastly, our study’s endpoint, the leakage rate, was determined based on the final results of two endoscopies, which may have limitations as it does not guarantee 100% accuracy in detecting lesions without leakage.

## 5 Conclusion

In summary, Linked Color Imaging (LCI) demonstrates superior performance in enhancing the detection of both Early Gastric Cancer (EGC) and gastrointestinal metaplasia compared to White Light Imaging (WLI). Its ability to detect gastric mucosal lesions with heightened sensitivity and efficiency suggests that LCI could emerge as a pivotal alternative to WLI for EGC and gastric precancerous lesion screening and monitoring. Nonetheless, further validation through additional clinical studies is warranted to solidify its role in clinical practice.

## Data Availability

The original contributions presented in the study are included in the article/**Supplementary Material**. Further inquiries can be directed to the corresponding author.

## Supplementary material

Supplementary Figure 1 Funnel plot of literature included in LCI and WLI gastric early cancer detection rates.

Supplementary Figure 2 Assessment of LCI and WLI for the diagnosis of GEC Detection rate Inter-study sensitivity analysis plot.

Supplementary Figure 3 Deek’s chart for LCI diagnosis of GEC.

Supplementary Figure 4 LCI diagnostic GEC fagan plot.

Supplementary Figure 5 LCI and WLI GIM Funnel plot of literature included in detection rate.

Supplementary Figure 6 Assessment of LCI and WLI for diagnosis of GIM Detection rate Inter-study sensitivity analysis plot.

Supplementary Figure 7 LCI diagnosis of GIM Deek’s diagram of.

Supplementary Figure 8 WLI Diagnostic Deek’s Chart for GIM.

Supplementary Figure 9 Fagan plot of LCI diagnosis of GIM.

Supplementary Figure 10 Fagan plots of WLI diagnostic GIMs.

Supplementary Document S1 Literature retrieval strategy.
