# Supplementary figures and images for: Diagnostic accuracy of linked color imaging and white light imaging for early gastric cancer and gastrointestinal metaplasia: a systematic review and meta-analysis
Source: Front Oncol. 2024 Nov 15;14:1480651. doi: 10.3389/fonc.2024.1480651 (PMC11604575; doi:10.3389/fonc.2024.1480651)

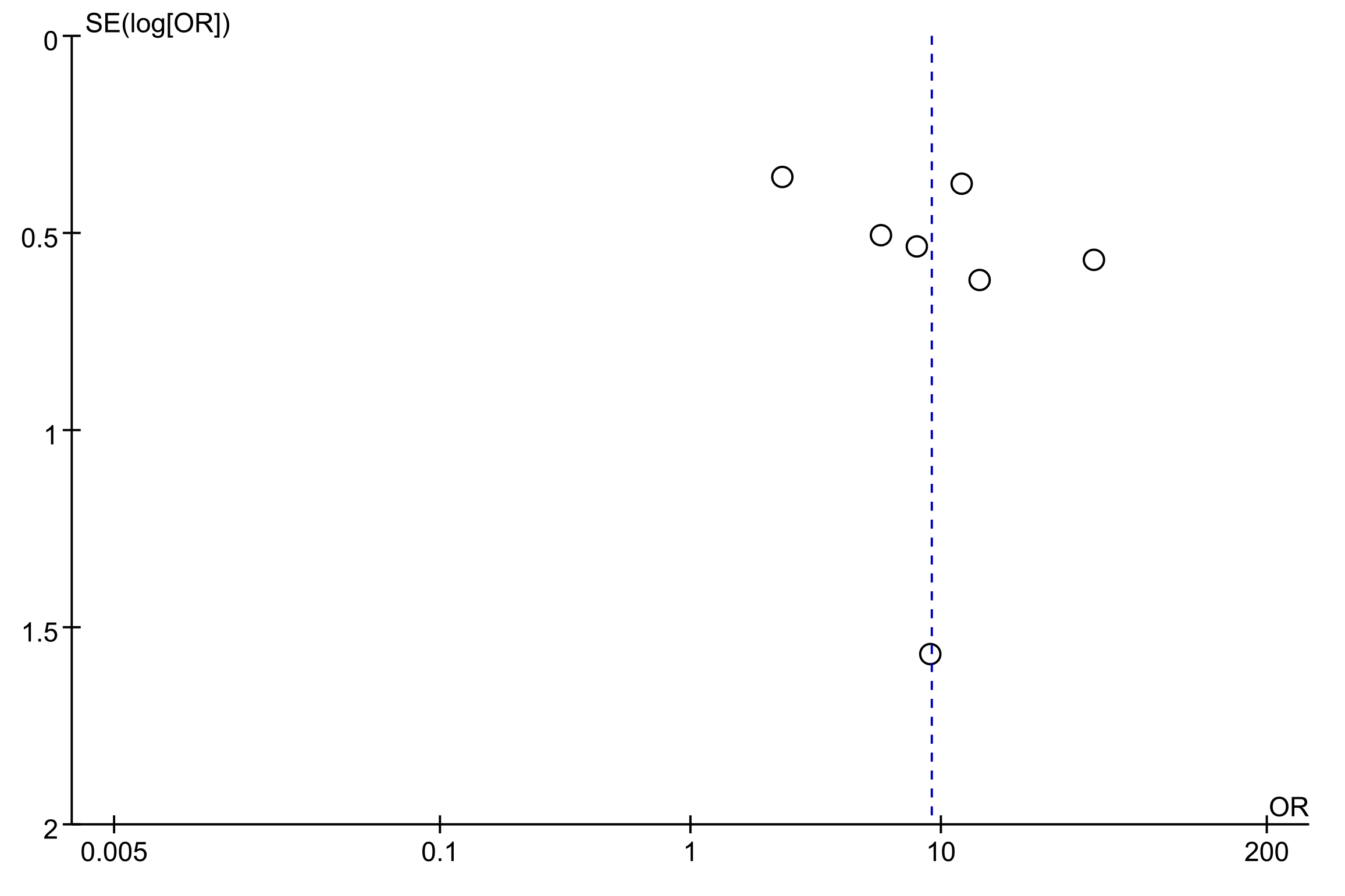

Supplement: Supplementary Figure 1 — Funnel plot of literature included in LCI and WLI gastric early cancer detection rates. [file Image1.tif]

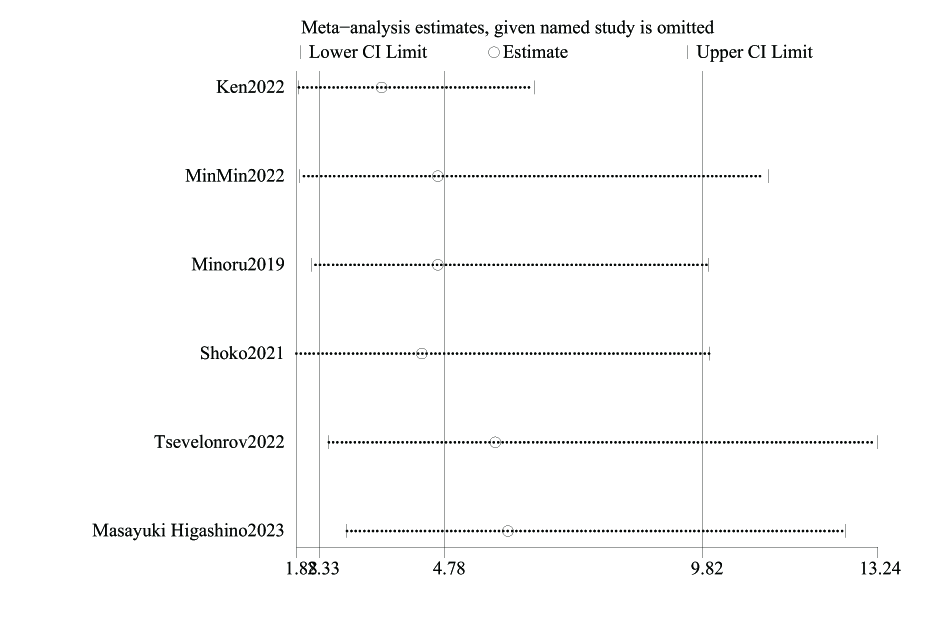

Supplement: Supplementary Figure 2 — Assessment of LCI and WLI for the diagnosis of GEC Detection rate Inter-study sensitivity analysis plot. [file Image2.tif]

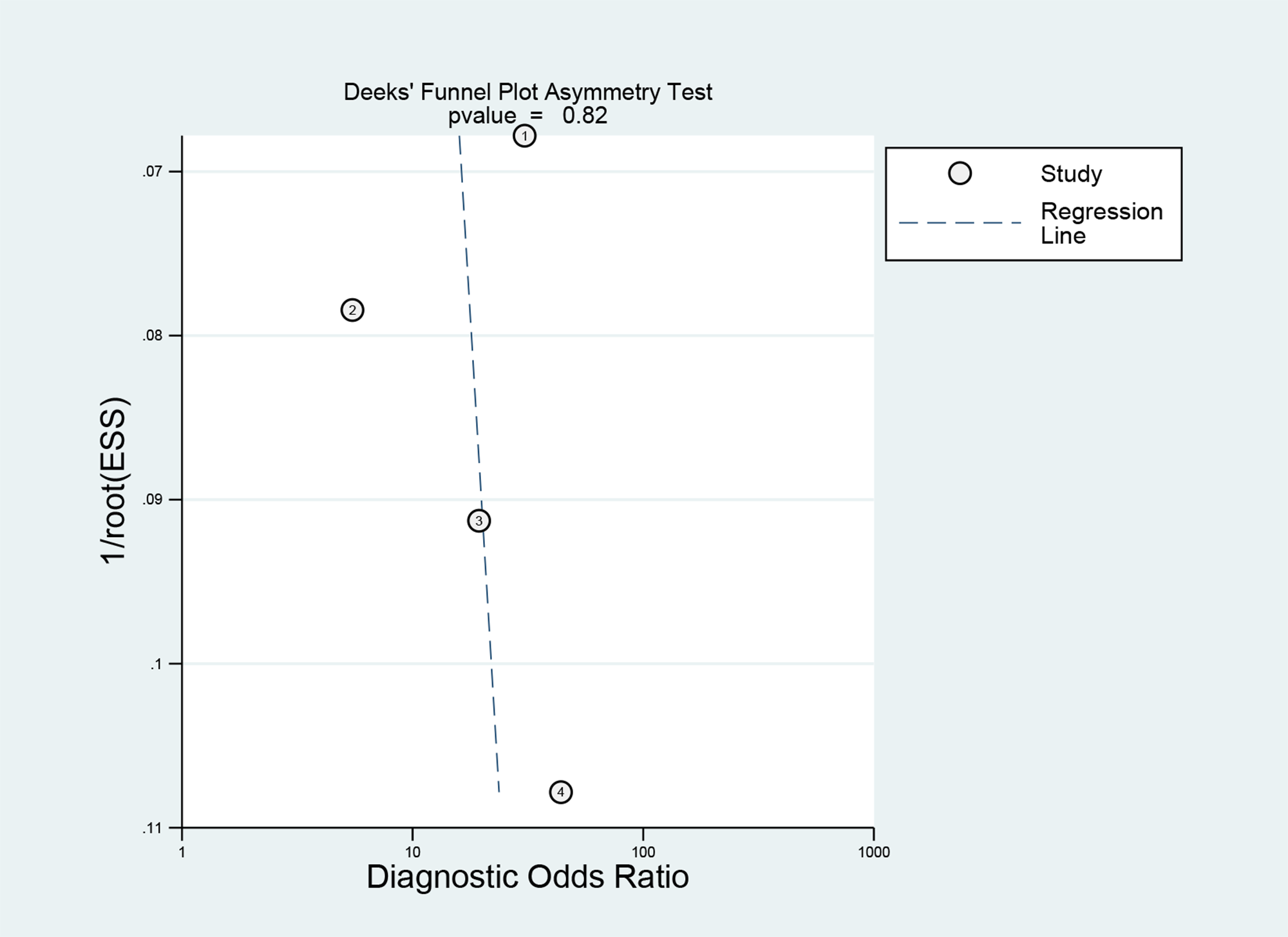

Supplement: Supplementary Figure 3 — Deek’s chart for LCI diagnosis of GEC. [file Image3.tif]

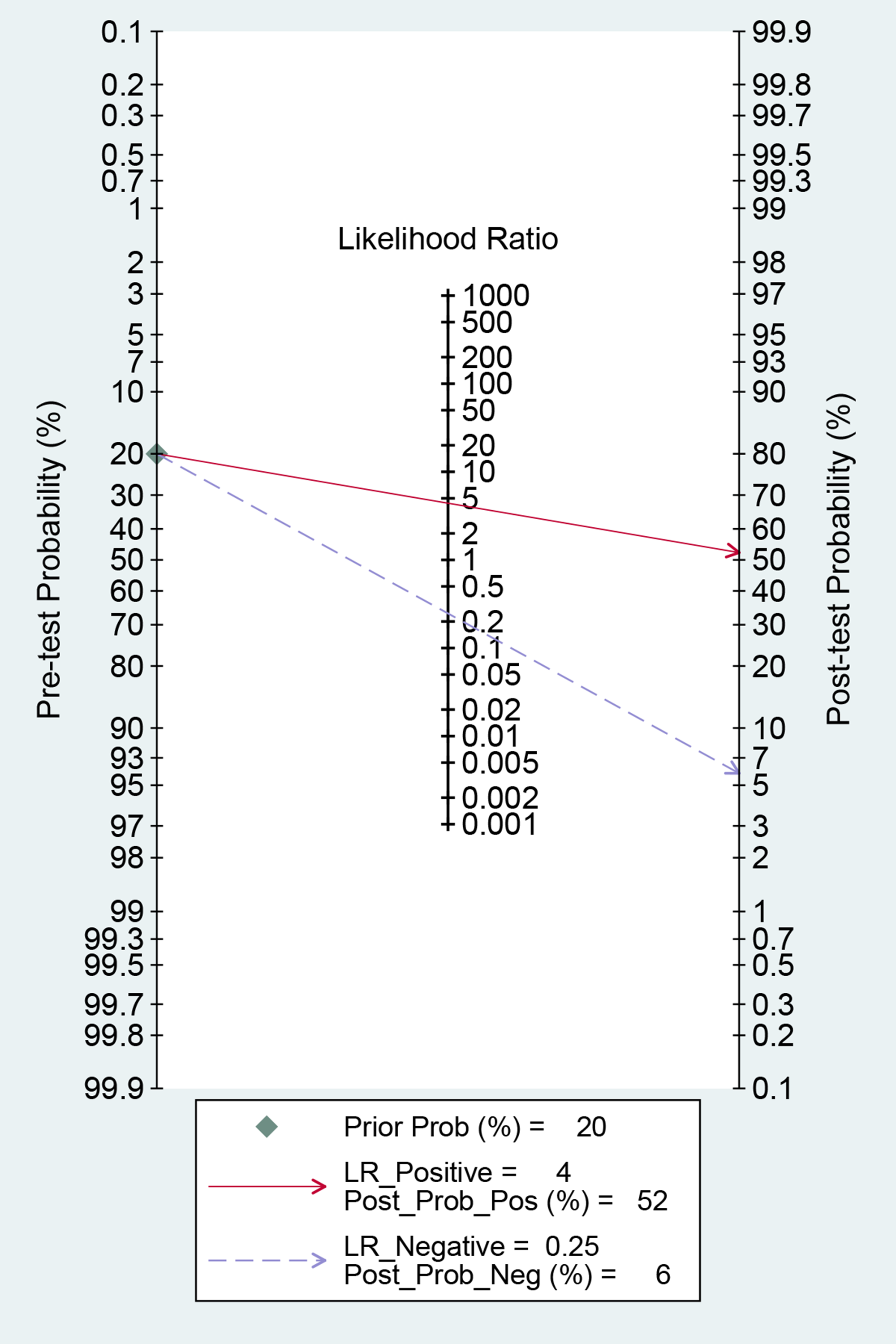

Supplement: Supplementary Figure 4 — LCI diagnostic GEC fagan plot. [file Image4.tif]

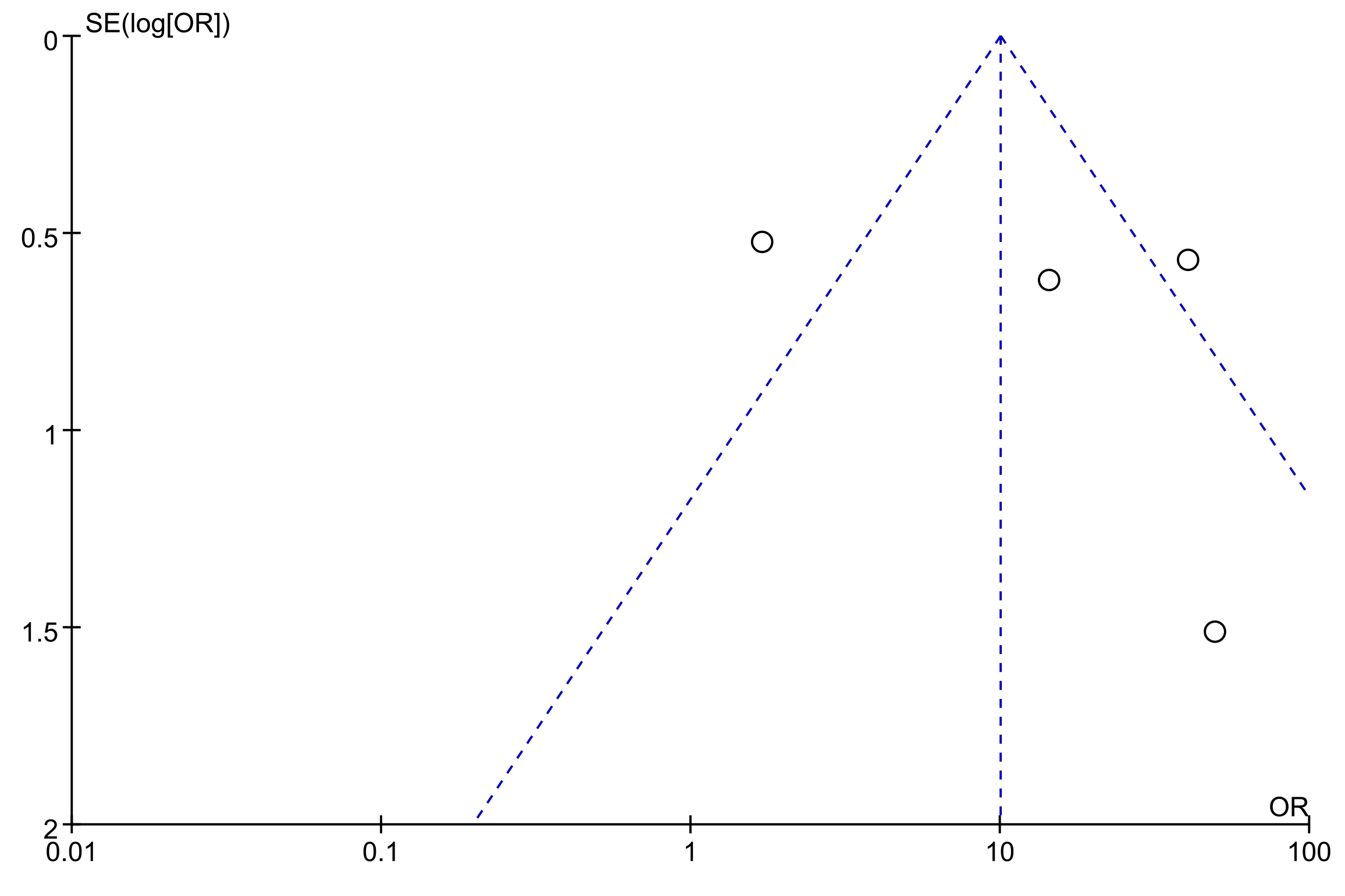

Supplement: Supplementary Figure 5 — LCI and WLI GIM Funnel plot of literature included in detection rate. [file Image5.tif]

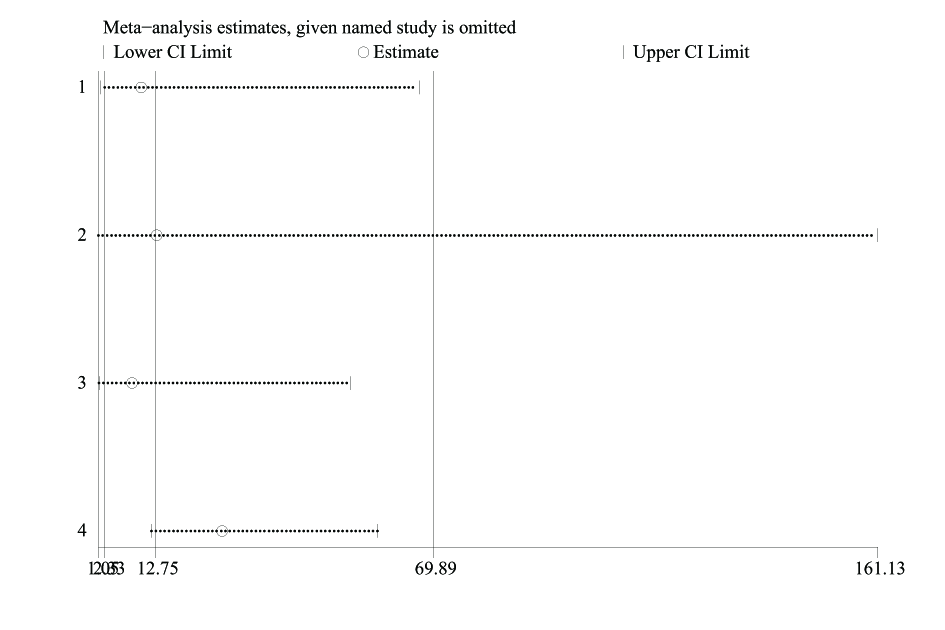

Supplement: Supplementary Figure 6 — Assessment of LCI and WLI for diagnosis of GIM Detection rate Inter-study sensitivity analysis plot. [file Image6.tif]

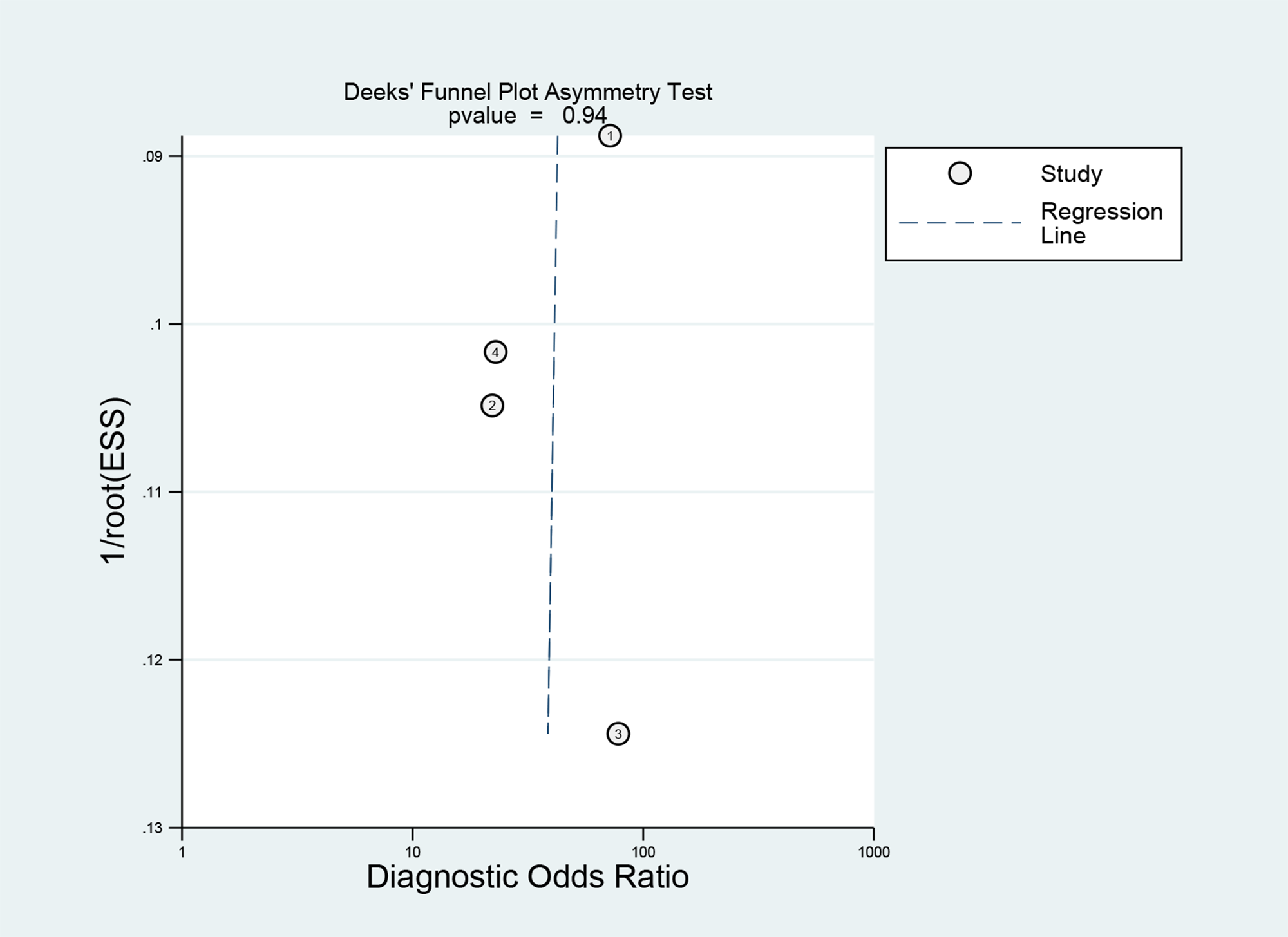

Supplement: Supplementary Figure 7 — LCI diagnosis of GIM Deek’s diagram of. [file Image7.tif]

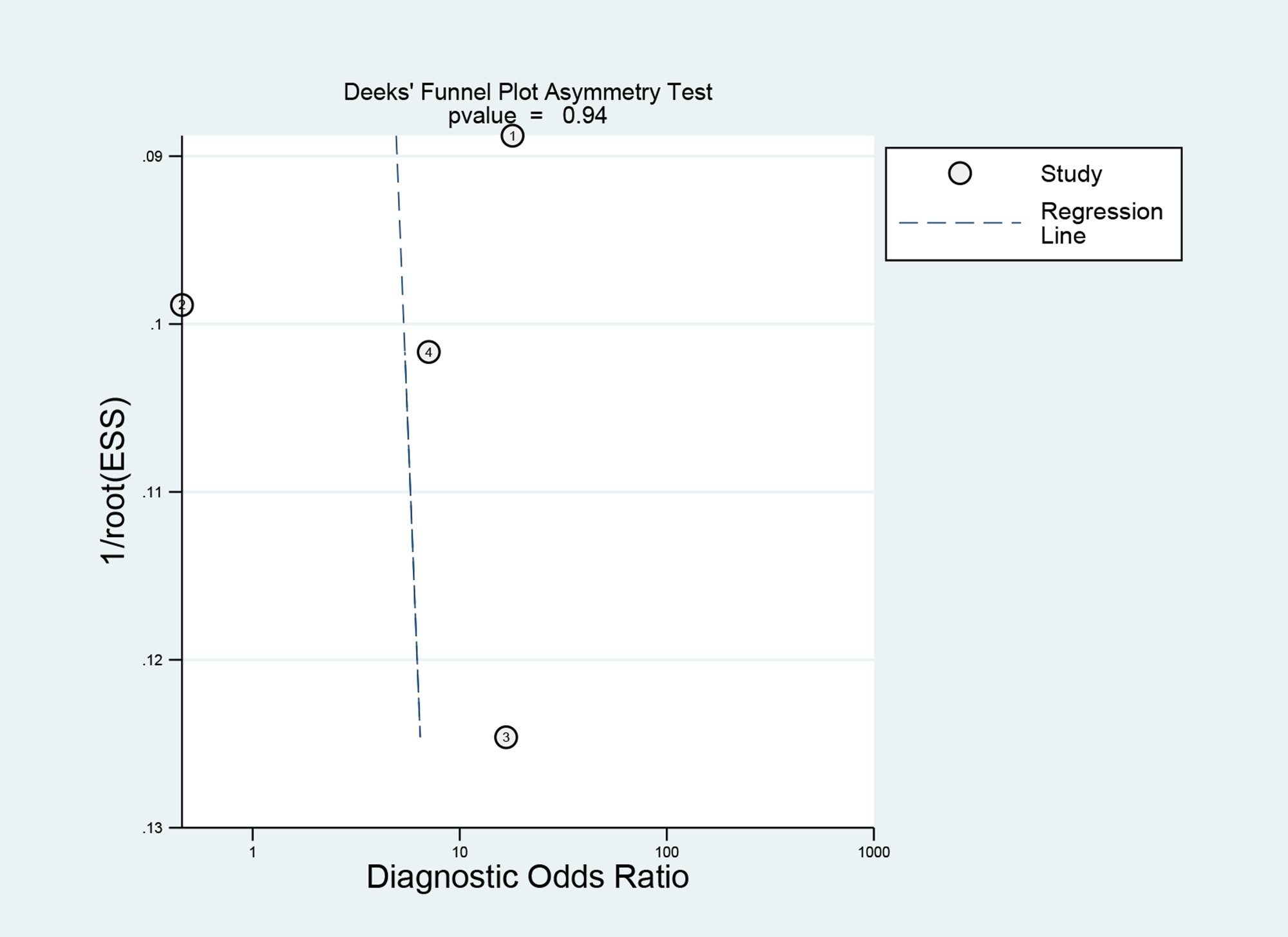

Supplement: Supplementary Figure 8 — WLI Diagnostic Deek’s Chart for GIM. [file Image8.tif]

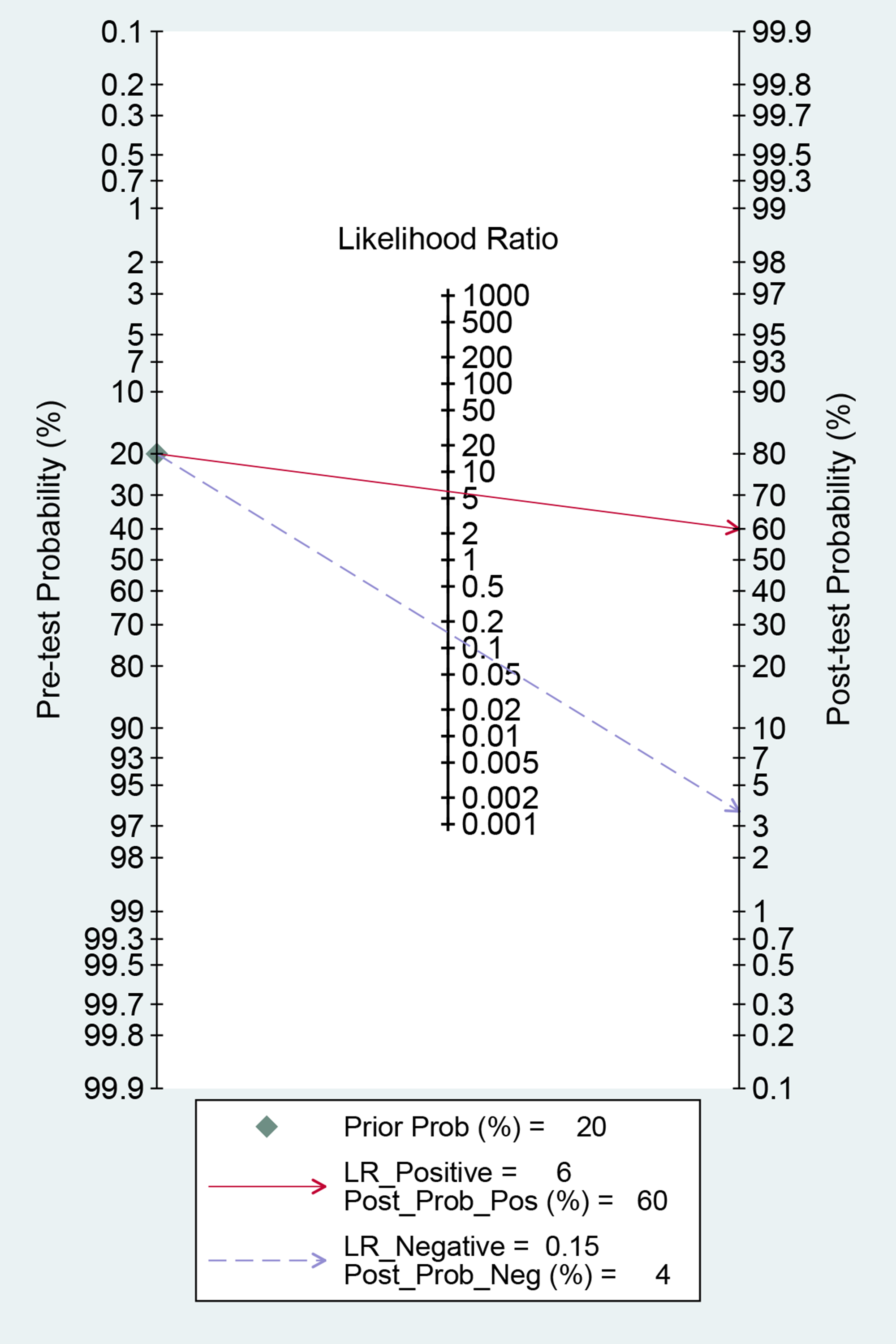

Supplement: Supplementary Figure 9 — Fagan plot of LCI diagnosis of GIM. [file Image9.tif]

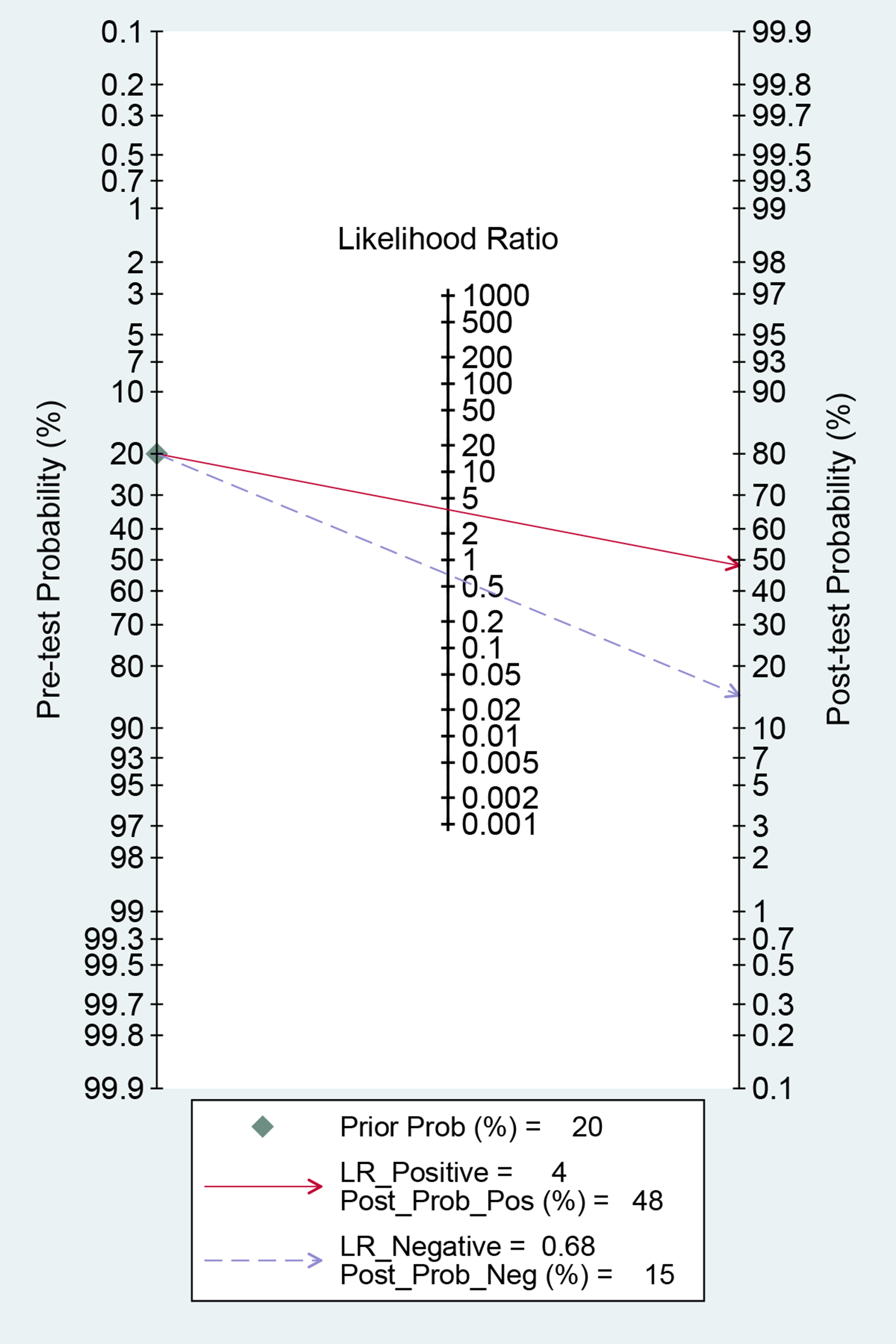

Supplement: Supplementary Figure 10 — Fagan plots of WLI diagnostic GIMs. [file Image10.tif]
