# Supplementary material for: Diagnostic accuracy of linked color imaging and white light imaging for early gastric cancer and gastrointestinal metaplasia: a systematic review and meta-analysis
Source: Front Oncol. 2024 Nov 15;14:1480651. doi: 10.3389/fonc.2024.1480651 (PMC11604575; doi:10.3389/fonc.2024.1480651)
Supplement: Supplementary Document S1 — Literature retrieval strategy. [file Table1.doc]

**Table 1：****Basic characteristics of the included literature and data extracted from the included studies（EGA Early gastric cancer）**

| Authors | Study period | Country | Inclusion of studies | Patients (n) | Indicators of outcome | |
| --- | --- | --- | --- | --- | --- | --- |
|  |  |  |  | LCI/WLI | LCI | WLI |
| Ken Haruma et al. | 2022 | Japan | Diagnostic ability of linked color imaging in ultraslim endoscopy to identify neoplastic lesions in the upper gastrointestinal tract | 751/753 | Detection rate89.32%（92/103） | Detection rate40.78%（42/103） |
| MinMin et al. | 2022 | China | Diagnostic accuracy of linked colour imaging versus white light imaging for early gastric cancers: a prospective, multicentre, randomized controlled trial study | 914/914 | 1.Sensitivity 89.47%  2.Specificity78.28 %  3.PPV 16.61%  4.NPV 99.35%  5.Detection rate 89.47%（51/57） | 1.Sensitivity 59.65 %  2.Specificity 68.95%  3.PPV 100%  4.NPV 96.71%  5.Detection rate 59.64%（34/57） |
| Minoru Yamaoka, et al. | 2019 | Japan | Detection of early stage gastric cancers in screening laser endoscopy using linked color imaging for patients with atrophic gastritis | 500/500 | Detection rate 100 %（13/13） | Detection rate 76.92%（10/ 13） |
| Shoko Ono et al. | 2021 | Janpa | Linked Color Imaging Focused on Neoplasm Detection in the Upper Gastrointestinal Tract | 751/751 | Detection rate 92. 31 %（60/65） | Detection rate 60%（36 /60 ） |
| Tsevelnorov Khurelbaatar et al. | 2022 | Janpa | Improved detection of early gastric cancer with linked color imaging using an ultrathin endoscop  e: a video-based analysis | 166/166 | 1.Sensitivity84.00 %  2.Specificity 50.60%  3.PPV 69.47%  4.NPV 70.27%  5.Detection rate 84.21 %（80/95） | 1.Sensitivity 69.90 %  2.Specificity59.8 %  3.PPV 69.94%  4.NPV 50.33%  5.Detection rate %（66 /95 ） |
| Masayuki Higashino | 2023 | Janpa | Improvement of detection sensitivity of upper gastrointestinal  epithelial neoplasia in linked color imaging based on data of  eye tracking | 120/120 | 1.Sensitivity68.1 %  2.Specificity90%  3.PPV 87.2%  4.NPV 73.9% | 1.Sensitivity53.7 %  2.Specificity86.7%  3.PPV 80.1%  4.NPV65.2% |
| Toshihisa Fujiyoshi | 2019 | Janpa | Utility of linked color imaging for endoscopic diagnosis of early gastric cancer | 43/43 | 1.Sensitivity 76.7 %  2.Specificity93.0%  3.PPV 91.7%  4.NPV 80%  5.Detection rate 76.7 %（33/43） |  |

**Table2：Basic characteristics of the included literature and data extracted from the included studies（GIM**

**Gastric Intestinal Metaplasia）**

| Authors | Study period | Country | Inclusion of studies | Patients (n) | Indicators of outcome | |
| --- | --- | --- | --- | --- | --- | --- |
|  |  |  |  | LCI/WLI | LCI | WLI |
| Clement | 2021 | Singapore | A prospective randomized tandem gastroscopy pilot study of linked color imaging versus white light imaging for detection of upper gastrointestinal lesions | 45/45 | 1.Sensitivity84.7 %  2.Specificity92.2 %  3.PPV76.6 %  4.NPV95.2 %  5.Detection rate 100 %（17 /17） | 1.Sensitivity 32.9 %  2.Specificity 97.2 %  3.PPV 77.8%  4.NPV 82.7 %  5.Detection rate 41.17 %（7 / 17） |
| Honglei Cheng et al. | 2019 | China | Predictability of gastric intestinal metaplasia by patchy lavender  color seen on linked color imaging endoscopy | 107/107 | 1.Sensitivity90.24%  2.Specificity72.72%  3.PPV67.27%  4.NPV92.31%  5.Detection rate 36.59%（33/37） | 1.Sensitivity36.59%  2.Specificity42.42%  3.PPV28.3%  4.NPV51.85%  5.Detection rate 89.18 %（15/41） |
| Shoko Ono et al. | 2018 | Janpa | Lavender Color in Linked Color Imaging Enables Noninvasive Detection of Gastric Intestinal Metaplasia | 128/128 | 1.Sensitivity91.40 %  2.Specificity 87.10%  3.PPV85.45 %  4.NPV 92.44%  5.Detection rate91.38%（53/58） | 1.Sensitivity 21.20 %  2.Specificity 99.20%  3.PPV95.64 %  4.NPV60.31 %  5.Detection rate20.69 %（12/58 ） |
| Jiang Zhang Xiu | 2021 | China | Comparison between the Linked Color and White Light  Imaging Combined Score in the Evaluation of High-Risk  Population of Gastric Cancer | 392/392 | 1.Sensitivity 80.7 %  2.Specificity 85.3 %  3.PPV 79.3%  4.NPV 86.4 % | 1.Sensitivity 69.6 %  2.Specificity 74.9 %  3.PPV 61.5%  4.NPV 82.4 % |
